# Relationship between the number of family members and stress by gender: Cross-sectional analysis of the fifth Korea National Health and Nutrition Examination Survey

**DOI:** 10.1371/journal.pone.0184235

**Published:** 2017-09-08

**Authors:** Jin-Won Noh, Kyoung-Beom Kim, Jumin Park, Janghun Hong, Young Dae Kwon

**Affiliations:** 1 Department of Healthcare Management, Eulji University, Seongnam, Korea; 2 Global Health Unit, Department of Health Sciences, University Medical Centre Groningen, University of Groningen, Groningen, the Netherlands; 3 Graduate School of Public Health, Korea University, Seoul, Korea; 4 National Institutes of Health Clinical Center, Bethesda, Maryland, United States of America; 5 Department of Humanities and Social Medicine, College of Medicine and Catholic Institute for Healthcare Management, the Catholic University of Korea, Seoul, Korea; National Institute of Health, ITALY

## Abstract

**Background:**

Due to gendered inequalities in the division of domestic work, women with paid employment and family caregiving responsibilities can feel extremely tired with general distress and depression. Therefore, the purpose of this study was to examine the association between the number of family members and stress level by gender among Korean adults using a nationally representative dataset.

**Methods:**

We used a sample of 6,293 subjects aged 19 or older (3,629 female and 2,264 male) from the fifth Korea National Health and Nutrition Examination Survey. A multivariable logistic regression analysis with sociodemographic and health-related characteristics was conducted. Because there were gender differences, a stratified analysis was performed for each gender.

**Results:**

Age, number of family members, education level, occupational status, depression, self-rated health status, and chronic diseases were found to have a significant association with stress level in the study subjects (*p*<0.05). The probability of perceiving stress increased among females from family with two members (OR 1.521), three family members (OR 1.893), or four or more family members without spouse (OR 2.035) compared to those who live alone.

**Conclusion:**

We found that unmarried women are more likely to be stressed as the number of family members increases. Gender expectations giving women the main responsibility for domestic and care work may become a source of stress. Reconciliation of family and work remains women’s responsibility in Korea. As family problems are recently becoming a big issue, our study shows the importance of considering gender difference in studies on stress according to family roles and functions.

## Introduction

In modern society, people take on multiple roles simultaneously such as a daily role of a family member, an informal care-giver role to family members who need help, an occupational role as a worker, and a volunteer role as a community member. In general, having multiple roles can create role conflict, leading to an increased risk of psychological distress such as stress [1–4]. For example, participants with childrearing or elderly caregiving responsibilities reported higher psychological distress compared to those with only an employment role [5]. Takeda et al found a positive relationship between the number of family members and worries [6]. Men in a nuclear family (i.e., married couple with children only) had significantly higher levels of worries about work, human relationships, and finances than those living only with their spouse. For women, respondents living in multigenerational households with their in-laws, or sandwiched between in-laws and their own children, expressed higher levels of worries about caregiving and human relationships compared with those living alone or living only with their spouse.

Different gender roles in work and family life influence the levels of psychological well-being [7,8]. Women were not part of the regular workforce until the mid-20th century because of traditional gender norms with regards to male breadwinning and female homemaking. For many industrialized nations including Korea, the role of women was viewed as being limited to household matters, while employment of men was recognized as central in their families’ lives [9]. As of the second half of the twentieth century, however, the proportion of female workers is increasing worldwide [10]. Despite increasing participation in paid employment, women continue to provide a much greater proportion of rearing children, serving as the communication hub for family members as well as for relatives, and managing family health [11,12]. Indeed, men have not increased their participation in the domestic sphere as much as women have in paid employment. In this regard, women might be more vulnerable to stress in both the home and workplace than men.

Despite the contingent nature of women’s participation in the labor force as well as the phenomenal changes in gender role expectations, few studies in Korea and Asia have explored gender differences on stress in families. A national study in the Republic of Korea may find interesting differences and similarities compared to studies published in the West. Therefore, this study aims to investigate gender differences in the relationship between stress level and number of family members, which is a proxy measure for the number of family roles, using a nationally representative dataset.

## Methods

### Data and subjects

We used data from the fifth Korea National Health and Nutrition Examination Survey (KNHANES) 2012, which are obtained from a public repository (http://knhanes.cdc.go.kr) without any restrictions. Data are available from the KNHANES database (https://knhanes.cdc.go.kr/knhanes/eng/sub03/sub03_02_02.do). The KNHANES is a nationwide, population-based, cross-sectional health survey and an ongoing surveillance system in Korea. This survey was conducted by the Ministry of Health and Welfare, and the Korea Centers for Disease Control and Prevention. Each survey year includes a new sample of approximately 10,000 individuals aged one year and over. A stratified multistage probability sampling design was used. To assure an equal probability of being sampled, weightings were assigned to each respondent. In the KNHANES 2012, there were a total of 8,058 samples. We excluded 1,765 individuals younger than 19 years for a study sample size of 6,293 individuals aged 19 years or older.

This study was approved by the Institutional Review Board of the Catholic University of Korea (MC15EISI0012) with a waiver for written informed consent because the data were obtained from a public repository and analyzed anonymously.

### Variables and measurement

Stress level was used as the outcome variable. In the survey, participants were asked about their experiences with stress, “How much stress do you feel in a day?”, and responded using a four-point scale corresponding to ‘none’, ‘some stress’, ‘high stress’, and ‘very high stress’. We classified responses into ‘low level (none or some stress)’ or ‘high level (high or very high stress)’.

Independent variables included sociodemographic and health-related factors that were indicated by prior literature and available in the KNHANES. Indicators of sociodemographic status are associated with stress [13–16]. In the current study, sociodemographic variables included sex, age, number of family members, education level, and occupational status. Age was treated as a continuous variable. The number of family members was classified into seven categories; ‘single’, ‘two members (without a spouse)’, ‘two members (with a spouse)’, ‘three members (without a spouse)’, ‘three members (with a spouse)’, ‘four members or more (without a spouse)’, or ‘four members or more (with a spouse)’. The term ‘spouse’ was applied to individuals who were legally cohabiting or married, while ‘single' was applied to widowed, divorced, separated, or unmarried individuals. Education level was classified into four categories (elementary school graduate or lower, junior high school graduate, high school graduate, or college graduate or higher). Occupational status was classified as ‘yes’ or ‘no’, with criteria for determining employed status as: (1) worked more than one hour with the intention of making a profit or engaged more than 18 hours as an unpaid family worker during the past week, or (2) persons with a job but who are temporarily absent. It is known that stress perception is associated with health-related variables such as presence of chronic diseases, depression, and self-rated health status [13,14,16]. In this study, presence of chronic diseases and depression was determined by physician’s diagnosis. Chronic diseases were defined as hypertension, dyslipidemia, stroke, myocardial infarction, angina pectoris, osteoarthritis, rheumatoid arthritis, pulmonary tuberculosis, asthma, diabetes mellitus, thyroid cancer, atopic dermatitis, renal failure, hepatitis types B and C, and liver cirrhosis. We counted each disease at the individual level and classified them into ‘no’ or ‘yes (having at least one kind of disease)’. Self-rated health status was reported on a five-point Likert scale and categorized as ‘(very) good’, ‘fair’, or ‘(very) poor’.

### Statistical analysis

Descriptive statistics are presented as the mean with standard deviation or the number with percentage. A univariable analysis, either a t or chi-squared test according to variable type, was performed to determine the difference in distribution of sociodemographic characteristics and health-related factors between sexes. Because there were gender differences, a stratified analysis was performed for each gender. In order to identify the factors associated with perceived stress based on gender after adjusting for the control variables, multivariable logistic regression analyses were performed for each sex. The variance inflation factor was calculated to assess multicollinearity in the logistic regression model. All statistical analyses were performed using Stata 14.2 (StataCorp LP, College Station, Texas, USA). The threshold for significance was set at 0.05 and was two-tailed.

## Results

The 6,293 subjects were composed of 2,664 males (42.3%) and 3,629 females (57.7%). In univariable analysis, most of the independent variables (i.e., number of family members, education level, occupational status, depression, self-rated health status, and chronic diseases) showed significantly varied distribution between the sexes (*p*<0.05), except for age. The percentage of single family member was 5.2% for males and 10.7% for females, which was approximately two times higher than males. The percentage of two member families not including a spouse was 4.8%, being 5.7% for female and 3.6% for males. As for three member families not including a spouse, the percentage was 6.8%, female being 6.8% and male 6.8%. Four member families not including a spouse, the percentage was 9.1%, female being 10.6% and male 6.9%. For perceived stress level, 28.2% of females reported feeling high stress, which was a greater percentage than males (21.4%, *p*<0.001) (Table 1).

**Table 1:** Characteristics of the study population and univariable analysis on stress level.

Variables	Subcategory	Total^a^	Female^b^	Male^c^	t or chi^2^ statistics
		n (%)	n (%)	n (%)	
Age	(Mean ± SD)	51.6 ± 17.0	51.4 ± 17.2	51.8 ± 16.7	0.691
Family members	Single	525 (8.4)	387 (10.7)	138 (5.2)	125.879**
	2 members (without a spouse)	302 (4.8)	207 (5.7)	95 (3.6)	
	2 members (with a spouse)	1,486 (23.7)	762 (21.1)	724 (27.3)	
	3 members (without a spouse)	428 (6.8)	247 (6.8)	181 (6.8)	
	3 members (with a spouse)	1,208 (19.3)	665 (18.4)	543 (20.5)	
	4 members or more (without a spouse)	568 (9.1)	384 (10.6)	184 (6.9)	
	4 members or more (with a spouse)	1,754 (28.0)	967 (26.7)	787 (29.7)	
Education level	Elementary or lower	1,443 (25.8)	1,029 (31.6)	414 (17.9)	132.914**
	Junior high school	601 (10.8)	342 (10.4)	259 (11.2)	
	High school	1,871 (33.5)	1,034 (31.5)	837 (36.2)	
	College or higher	1,677 (30.0)	877 (26.7)	800 (34.6)	
Occupational status	No	2,338 (41.8)	1,719 (52.3)	619 (26.8)	363.024**
	Yes	3,256 (58.2)	1,566 (47.7)	1,690 (73.2)	
Depression	No	5,352 (95.4)	3,083 (93.6)	2,269 (98.0)	60.711**
	Yes	257 (4.6)	211 (6.4)	46 (2.0)	
Self-rated health	Good	1,686 (30.1)	878 (26.6)	808 (34.9)	58.057**
	Fair	2,848 (50.7)	1,702 (51.6)	1,144 (49.4)	
	Poor	1,079 (19.2)	716 (21.7)	363 (15.7)	
Chronic diseases	No	2,775 (49.5)	1,588 (48.2)	1,187 (51.3)	5.111*
	Yes	2,834 (50.5)	1,706 (51.8)	1,128 (48.7)	
Stress level	Low	4,167 (74.6)	2,353 (71.8)	1,814 (78.6)	32.857**
	High	1,420 (25.4)	925 (28.2)	495 (21.4)	

*p<0.05.

**p<0.001.

^a^. sample size = 6,293.

^b^. sample size = 3,629.

^c^. sample size = 2,664.

SD, standard deviation; ref, reference.

The result of the multivariable analysis based on gender was significantly associated with age, occupational status, depression, and self-rated health status in both sexes. Higher age was found to be related to lower risk of perceiving stress in both females (OR 0.984) and males (OR 0.972). In the case of occupational status, employed subjects showed a higher risk of perceiving stress in both females (OR 1.282) and males (OR 1.508). The proportion perceiving stress was higher among depressed individuals in both females (OR 2.032) and males (OR 3.771). Self-rated health status was negatively associated with a higher risk of perceiving stress in both sexes. Number of family members and education level significantly affected stress level only among females. The perceived stress among female without spouse increased from those living in a household with two-family members (OR 1.521), three-family members (OR 1.893), or four or more-family members (OR 2.035) as compared to those who were living alone. Female living with a spouse showed a higher risk of perceiving stress (two-person family OR 1.153; three-person family OR 1.258; four or more-person family OR 1.344) compared to those who live alone, but which were not statistically significant. Stress was significantly decreased for those who had a junior high school (OR 0.581) education compared to the elementary or lower group only among females (Table 2).

**Table 2:** Multivariable logistic regression analysis on perceived stress by gender.

Variables	Subcategory	Female			Male		
		OR	95% CI		OR	95% CI	
Age	(min = 19, max = 98)	0.984***	0.976	0.992	0.972***	0.961	0.982
Family members	Single	ref			ref		
	2 members (without a spouse)	1.521*	0.995	2.324	0.713	0.326	1.558
	2 members (with a spouse)	1.153	0.838	1.586	1.173	0.685	2.010
	3 members (without a spouse)	1.893**	1.252	2.863	0.904	0.479	1.704
	3 members (with a spouse)	1.258	0.895	1.768	1.157	0.678	1.975
	4 members or more (without a spouse)	2.035***	1.389	2.982	1.144	0.609	2.152
	4 members or more (with a spouse)	1.344*	0.969	1.866	1.494	0.895	2.493
Education level	Elementary or lower	ref			ref		
	Junior high school	0.581**	0.423	0.797	1.089	0.704	1.685
	High school	0.765*	0.578	1.011	0.928	0.643	1.338
	College or higher	0.960	0.707	1.303	1.367*	0.950	1.968
Occupational status	No	ref			ref		
	Yes	1.282**	1.088	1.511	1.508**	1.137	1.999
Depression	No	ref			ref		
	Yes	2.032***	1.503	2.747	3.771***	2.023	7.029
Self-rated health	Good	Ref			ref		
	Fair	1.845***	1.495	2.277	1.655***	1.289	2.123
	Poor	4.958***	3.828	6.421	4.442***	3.187	6.192
Chronic diseases	No	ref			ref		
	Yes	0.926	0.763	1.123	0.902	0.710	1.146

*p<0.1.

**p<0.01.

***p<0.001.

OR, odds ratio; CI, confidence intervals; ref, reference.

## Discussion

This study analyzed the association between the number of family members and the level of stress by examining a sample of 2,664 men and 3,629 women aged 19 to 98 extracted from the fifth KNHANES, controlling for main socio-demographic and health-related variables by gender. In the case of men, the association between the number of family members and the level of stress was not statistically significant; however, for women, the level of stress increased in proportion to the number of family members. This confirms that the more family members there are, or in other words, the more roles there are that need to be performed within the family, the more likely that women will experience greater stress.

In the past, for many industrialized nations including Korea women were considered physically and morally unsuitable for wage labor and thus were required to fulfill housekeeping, childcare, and family care responsibilities, with laws and custom limiting women’s wage labor [17]. In modern societies, more women are participating in paid employment, but they also perform an average of 31.5 hours per week of unpaid work, whereas men spend only 21 hours per week on unpaid work. This demonstrates that women still carry the bulk of housekeeping work, and excessive unpaid labor decreases recreational time, which negatively affects one’s wellbeing [18]. The more women concentrate on childcare, the more stressed they become in comparison to men [19]. An overload of roles from extensive childcare and care for other family members may cause stress, depression, and extreme fatigue in women [20].

These women-specific problems can be seen as a result of conflict between the increased economic role of women in modern societies and the traditional social expectations that require women to fulfill homemaking duties [21]. An increase in family members means an increase in people to look after, which means an increased burden for women who are the main caregivers for family members, and this can increase stress in women. Unless there are changes in the common social notion that takes women’s roles as homemakers and caretakers for granted including changes in expectations regarding Korean husbands and fathers and their actual behavior, the correlation between an increase in family members and women’s stress level will remain unchanged.

Aside from the number of family members, variables such as age, occupational status, depression, and self-rated health status were shown to be statistically associated with stress for both genders in this study. Marital status and education level were significant only for women, which coincides with the results of most previous studies.

Both men and women appeared to be at a lower risk of stress as they age. It has been reported that stress has less of an impact on older people than on younger people [22]. Economically active men and women were more likely to be under higher stress than men and women who were not economically active. Many adults spend most of their time in two areas, at work or with family, and fulfilling both professional and household work duties at the same time can be a source of potential stress [23]. In this study, both men and women with depression experienced more stress, which coincides with the results of a previous study that found a strong correlation between stress and depression [24]. In the current study, both men and women were more likely to have more stress if they assessed their own health status more negatively, e.g., participants who assessed their health as ‘fair’ were more likely to have a higher level of stress than those who assessed their health as ‘good’, and those who assessed their health as ‘poor’ were more likely to have a higher level of stress than with ‘fair’ health. Another previous study has also shown that people who assessed their health status as ‘excellent’ or ‘good’ had less mental stress, depression, or other general symptoms of anxiety [25].

Married women had less risk of stress than women without spouses. It has been known that married women have less stress since their husbands can provide emotional support [26]. In regard to education level, participants with a higher level of education had less stress, not including university graduates. It has been reported that people with higher education levels have more control over their lives and a lower level of perception of their own stress [27], but there are insufficient studies to accurately explain the correlation between education level and stress by gender.

This study has a few limitations. It measured stress level based on a question asking about the participant’s perceived stress level and sorted the answers into two categories (i.e., ‘high’ or ‘low’); therefore, this study cannot sufficiently reflect quantitative differences in stress levels. This study only considered the quantitative aspects of family roles and functions using the number of family members as a variable. Future studies should consider qualitative aspects of family roles and functions, as well as different types of families such as single-parent families, grandparent-headed families, extended families, and nuclear families. This study is a cross-sectional study, which only shows the association between the number of family members and stress and not the chronological relationship. In the future, studies that show the cause-effect relationship through time series analysis are needed.

Despite these limitations, this study is significant in that it confirmed gender differences in the correlation between stress and number of family members for the first time using nationally representative data. This study is also important as most studies on family stress by gender focus on developed Western countries, with a limited number of in-depth studies on Asian countries. Additional comparative studies with other cultures are needed to generalize study results.

## Conclusions

In the transition from a traditional, male-dominant society to modern times where women have increased opportunity for social activity, women were faced with a conflict that called for fulfilling professional and household work functions at the same time, which became a source of stress for women. This study analyzed South Korea’s nationally representative data and confirmed that more family members may contribute to a higher level of stress for women. This shows the importance of considering gender difference in studies on stress by family roles and functions.
